# Whole-Genome Sequencing and Comparative Genomics Analysis of a Newly Emerged Multidrug-Resistant Klebsiella pneumoniae Isolate of ST967

**DOI:** 10.1128/spectrum.04011-22

**Published:** 2023-04-06

**Authors:** Jie Sheng, Rory Cave, Mary M. Ter-Stepanyan, Nune Kotsinyan, Jiazhen Chen, Li Zhang, Taijiao Jiang, Hermine V. Mkrtchyan

**Affiliations:** a Institute of Systems Medicine, Chinese Academy of Medical Sciences, Peking Union Medical College, Beijing, China; b Suzhou Institute of Systems Medicine, Suzhou, China; c Institute of Basic Medical Sciences, Chinese Academy of Medical Sciences, School of Basic Medicine, Peking Union Medical College, Beijing, China; d School of Biomedical Sciences, University of West London, London, United Kingdom; e Yerevan State Medical University after Mkhitar Heratsi, Faculty of Public Health, Department of Epidemiology, Yerevan, Republic of Armenia; f Research Center of Maternal and Child Health Protection, Yerevan, Armenia; g National Centre for Disease Control and Prevention, Yerevan, Armenia; h Department of Infectious Disease, Huashan Hospital, Fudan University, Shanghai, China; i Guangzhou Laboratory, Guangzhou, China; Brown University

**Keywords:** *Klebsiella pneumoniae*, whole-genome sequencing, multidrug resistance, phylogenetic analysis

## Abstract

Klebsiella pneumoniae is a common cause of hospital- and community-acquired infections globally, yet its population structure remains unknown for many regions, particularly in low- and middle-income countries (LMICs). Here, we report for the first-time whole-genome sequencing (WGS) of a multidrug-resistant K. pneumoniae isolate, ARM01, recovered from a patient in Armenia. Antibiotic susceptibility testing revealed that ARM01 was resistant to ampicillin, amoxicillin-clavulanic acid, ceftazidime, cefepime, norfloxacin, levofloxacin, and chloramphenicol. Genome sequencing analysis revealed that ARM01 belonged to sequence type 967 (ST967), capsule type K18, and antigen type O1. ARM01 carried 13 antimicrobial resistance (AMR) genes, including *bla*_SHV-27_, *dfrA12*, *tet*(A), *sul1*, *sul2*, *catII.2*, *mphA*, *qnrS1*, *aadA2*, *aph3-Ia*, *strA*, and *strB* and the extended-spectrum β-lactamase (ESBL) gene *bla*_CTX-M-15_, but only one known virulence factor gene, *yagZ*/*ecpA*, and one plasmid replicon, IncFIB(K)(pCAV1099-114), were detected. The plasmid profile, AMR genes, virulence factors, accessory gene profile, and evolutionary analyses of ARM01 showed high similarity to isolates recovered from Qatar (SRR11267909 and SRR11267906). The date of the most recent common ancestor (MRCA) of ARM01 was estimated to be around 2017 (95% confidence interval [CI], 2017 to 2018). Although in this study, we report the comparative genomics analysis of only one isolate, it emphasizes the importance of genomic surveillance for emerging pathogens, urging the need for implementation of more effective infection prevention and control practices.

**IMPORTANCE** Whole-genome sequencing and population genetics analysis of K. pneumoniae are scarce from LMICs, and none has been reported for Armenia. Multilevel comparative analysis revealed that ARM01 (an isolate belonging to a newly emerged K. pneumoniae ST967 lineage) was genetically similar to two isolates recovered from Qatar. ARM01 was resistant to a wide range of antibiotics, reflecting the unregulated usage of antibiotics (in most LMICs, antibiotic use is typically unregulated.) Understanding the genetic makeup of these newly emerging lineages will aid in optimizing antibiotic use for patient treatment and contribute to the worldwide efforts of pathogen and AMR surveillance and implementation of more effective infection prevention and control strategies.

## INTRODUCTION

Commonly associated with the environment (1), plants (2), animals (3, 4), and humans (5), Klebsiella pneumoniae has emerged as a major global public health threat due to becoming a leading cause of nosocomial and community infections over the past decade. This opportunistic pathogen, responsible for a wide range of infections (6–10), has also been associated with multidrug resistance (MDR). A recent survey conducted in 41 countries by the Central Asian and the European Surveillance of Antimicrobial Resistance network (11) revealed that >50% of the isolates recovered from patients (predominantly in Southern and Eastern Europe) were resistant to third-generation cephalosporins and >25% were resistant to carbapenems. Moreover, mortality rates caused by MDR K. pneumoniae have been reported to be as high as 50% or higher in clinical settings (12, 13). This is further exacerbated by new emerging clones, such as sequence types 101 (ST101), ST307, and ST147, which have acquired virulence factors and antimicrobial resistance (AMR) genes, contributing to their success as persistent nosocomial infections (14, 15). One of the key antibiotic resistance gene families found in extended-spectrum β-lactamase (ESBL)-producing K. pneumoniae isolates that have been dominating nosocomial infections globally is the *bla*_CTX-M-type_ (16, 17). In addition, *bla*_CTX-M-15_ has been identified as the most predominant type and has rapidly disseminated in K. pneumoniae isolates recovered from humans (18, 19) and animals (3, 20).

The driving force for the development of multidrug resistance is selective pressure, which is due to continuous exposure to multiple antibiotics in settings where they are overused and/or misused, such as health care and agriculture (21). In many low-and middle-income countries (LMICs), public exposure to MDR bacteria is increasingly high due to uncontrolled use of “antibiotic growth promoters” in animals and the ability to buy antibiotics “over-the-counter” (22). In addition, there are few clinical guidelines for the use of antibiotics for human treatment in animals. Moreover, severe financial constraints, a lack of diagnostic facilities, and inadequate pathogen surveillance systems make infection prevention and control (IPC) extremely challenging, which encourages the development of multidrug-resistant strains (23).

The spread of MDR K. pneumoniae is a global public health issue. Understanding the genetic makeup of new emerging lineages of such pathogens will enable us to optimize antibiotic use for patient treatment and contribute to the worldwide efforts of tackling antibiotic resistance and design appropriate IPC measures (24, 25). Whole-genome sequencing (WGS) has been widely used in studying the global transmission and molecular mechanisms of K. pneumoniae (5, 26, 27). However, reports on whole-genome sequencing and population genetics of K. pneumoniae from LMICs are scarce (28). A systematic review we conducted for Armenia, a low- and middle-income country, showed that only one study has analyzed secondary data on AMR for the period of 2016 to 2019 (29). We recently conducted a small-scale pilot study to identify the genetic diversity of methicillin-resistant Staphylococcus aureus (MRSA) in a teaching hospital in Armenia, which provided insights into a previously unrecognized diversity of MRSA clones in Armenia, including pandemic and sporadic lineages seen internationally (30). Further WGS studies identified a novel CC8 MRSA clone circulating in a hospital setting in Yerevan, the capital of Armenia. Cross transmission/contamination due to the failure of infection control and prevention strategies was evident as MRSA isolates belonging to the same lineages were recovered from patients and hospital environments (31).

Here, we report for the first time the whole-genome sequencing analysis of a K. pneumoniae isolate, ARM01, recovered from a patient in Armenia and provide insights into its genomic features. Further comparative genomics analysis revealed its temporal and spatial phylogenetic dynamics.

## RESULTS

### Antibiotic susceptibility testing and genomic features.

In total, eight K. pneumoniae isolates were received from the Medical Microbiology laboratories of three hospitals in Armenia between January 2019 and August 2019 (see Table S1 in the supplemental material). Whole-genome sequencing revealed that four out of eight isolates belonged to sequence type 307 (ST307), with other four isolates each belonging to ST37, ST147, ST807, and ST967. All isolates were resistant to ampicillin, amoxicillin-clavulanic acid, ceftazidime, and cefepime, but were sensitive to meropenem (Table 1). In addition to the above antibiotics, the ARM01 isolate belonging to ST967 described further in this study was also resistant to norfloxacin, levofloxacin, and chloramphenicol (Table 1).

**TABLE 1:** Antimicrobial susceptibility profiles of eight K. pneumoniae isolates

Isolate	Resistance or sensitivity to^*a*^:										
	AMP	PTZ	AMC	CAZ	CEP	NOR	LVX	AMK	IPM	MEM	CHL
ARM01	R	S	R	R	R	R	R	S	S	S	R
ARM02	R	S	R	R	R	S	S	S	S	S	S
ARM03	R	R	R	R	R	R	R	I	S	S	R
ARM04	R	I	R	R	R	R	R	S	I	S	R
ARM05	R	I	R	R	R	R	R	S	I	S	R
ARM06	R	R	R	R	R	R	R	S	S	S	R
ARM07	R	R	R	R	R	R	R	S	I	S	R
ARM08	R	S	R	R	R	R	R	S	S	S	S

a R, resistant; I, intermediate resistance; S, sensitive. AMP, ampicillin (10 mg); PTZ, piperacillin-tazobactam (30/6 mg); AMC, amoxicillin (20 mg)-clavulanic acid (10 mg); CAZ, ceftazidime (10 mg); CEP, cefepime (30 mg); NOR, norfloxacin (10 mg); LVX, levofloxacin (5 mg); AMK, amikacin (30 mg); IPM, imipenem (10 mg); MEM, meropenem (10 mg); CHL, chloramphenicol (30 mg).

Multilocus sequence typing (MLST) and serotype analysis revealed that K. pneumoniae ARM01 belonged to sequence type 967 (ST967), capsule type K18, and the O antigen type O1 genotype. Thirteen known AMR genes conferring resistance to nine classes of antibiotics were present in ARM01 (Table 2). These included trimethoprim resistance gene *dfrA12*, tetracycline resistance gene *tet*(A), sulfonamide resistance genes *sul1* and *sul2*, phenicol resistance gene *catII.2*, macrolide resistance gene *mphA*, fluoroquinolone resistance gene *qnrS1*, aminoglycoside resistance genes *aadA2*, *aph3-Ia*, *strA*, and *strB*, third-generation cephalosporin resistance gene *bla*_CTX-M-15_, and β-lactamase resistance gene *bla*_SHV-27_. Only one known virulence-associated gene, *yagZ*/*ecpA*, which encodes the common pilus fibrillin subunit, was found in ARM01. Moreover, ARM01 harbored one plasmid replicon, IncFIB(K)(pCAV1099-114).

**TABLE 2:** Genomic characteristics of ARM01^*a*^

Antibiotic, genetic element, or molecular data	Gene type or molecular data
Antimicrobial resistance	Resistance genes
Cephalosporins (3rd generation)	*bla*_CTX-M-15_
ESBLs	*bla*_SHV-27_
Trimethoprim	*dfrA12*
Tetracycline	*tet*(A)
Sulfonamides	*sul1*, *sul2*
Phenicols	*catII.2*
Macrolides	*mphA*
Fluoroquinolones	*qnrS1*
Aminoglycosides	*aadA2*, *aph3-Ia*, *strA*, *strB*
Virulence-associated genetic elements	Virulence-associated genes
Hypermucoidy (*rmpA* and/or *rmpA2*)^*b*^	−
Yersiniabactin (*ybt*)	−
Colibactin (*clb*)	−
Salmochelin (*iro*)	−
Aerobactin (*iuc*)	−
Others	*yagZ/ecpA*
Molecular data	
MLST	ST967
Capsule (K) serotype	K18
O antigen (LPS) serotype	O1
Plasmid replicon	IncFIB(K)(pCAV1099-114)
ICEs	−

a ESBLs, extended-spectrum-β-lactamases; ICEs, integrative and conjugative elements; −, negative.

b Hypermucoidy (*rmpA* and/or *rmpA2*) represents the regulator of mucoid phenotype A.

### Phylogenomic analysis of K. pneumoniae ARM01.

To investigate the ARM01 isolate more thoroughly, we genetically compared it with 22 publicly available K. pneumoniae ST967 isolates obtained from the Pathogenwatch database and previously published studies (32) (Table S2). A total of 23 K. pneumoniae ST967 isolates collected from 12 countries were included, comprising isolates recovered from humans (*n* = 21), river water (*n* = 1), and a broiler (*n* = 1). These isolates were identified as belonging to 3 different K (capsule) and O antigen (lipopolysaccharide [LPS]) serotypes.

**FIG 1:**
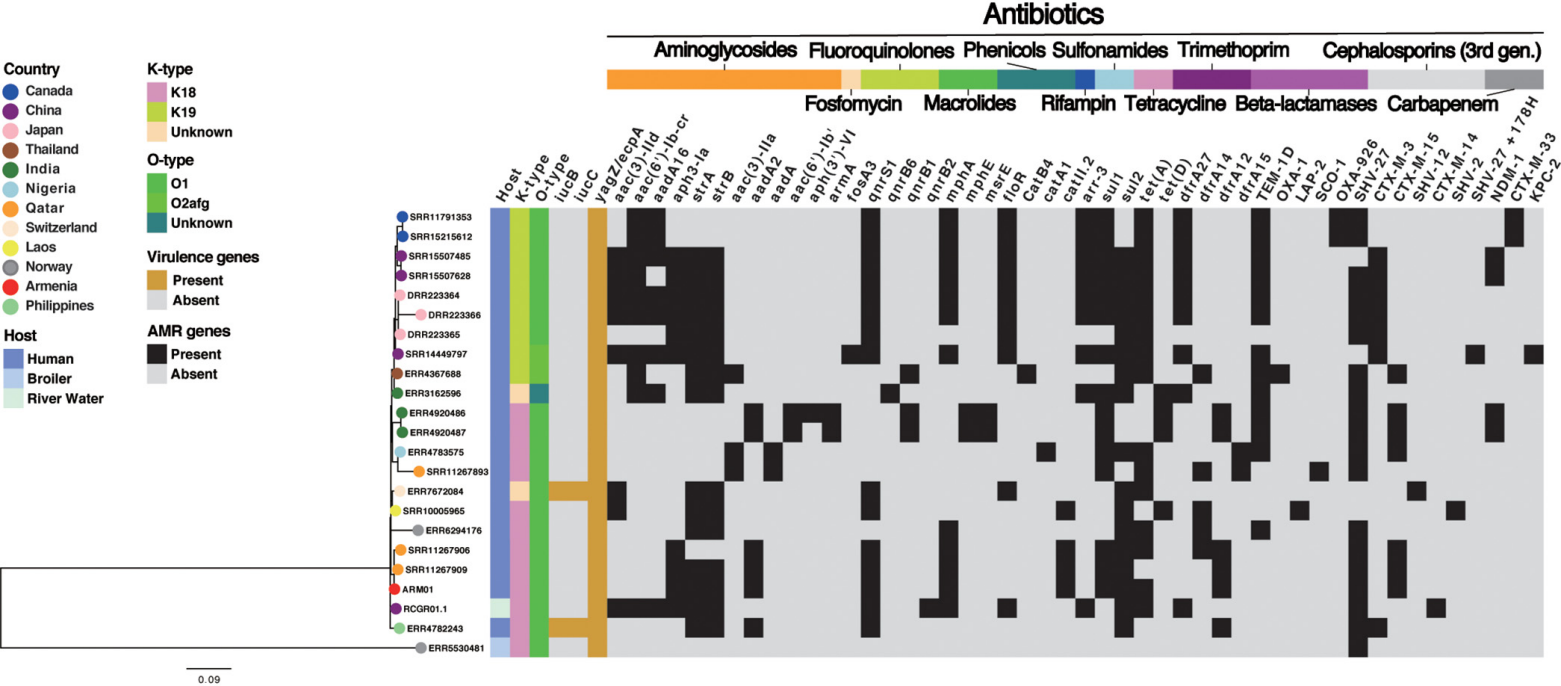
Core SNP phylogenetic analysis with virulence and AMR determinants found in 23 K. pneumoniae ST967 isolates. The color of the circle at the end of each branch represents the country where the isolate was collected. The heatmap columns are as follows: (i) host, (ii) capsule (K) serotype, (iii) O antigen (LPS) serotype, (iv) virulence determinants (orange, present; gray, absent), and (v) AMR determinants (black, present; gray, absent).

The core SNP phylogenetic tree showed that ARM01 was phylogenetically closely related to two human isolates recovered from Qatar (SRR11267906 and SRR11267909), forming a single monophyletic cluster (Fig. 1). Moreover, assessing the 3,474 accessory genes identified in the pangenome, we found that ARM01 shared many of the same accessory genes found in two isolates recovered from Qatar (*r* = 0.77 for SRR11267909 and *r* = 0.72 for SRR11267906) (see Fig. S1A in the supplemental material). The close genetic similarities of ARM01 and the two Qatar isolates (SRR11267906 and SRR11267909) were further confirmed based on genome-wide average nucleotide identity (ANI) analysis. The Hadamard values of ARM01 with SRR11267909 and SRR12267906 were 0.988 and 0.984, respectively (Fig. S1B and Table S3). In addition, hierarchical clustering of plasmid replicon patterns revealed that ARM01 shared the same replicon, IncFIB(K)(pCAV1099-114), with the Qatar isolates (Fig. S2 and Table S4). The above findings indicate that the Armenian isolate (ARM01) was genetically closely related to two Qatar isolates (SRR11267906 and SRR11267909).

### Evolutionary origins of K. pneumoniae ARM01.

**FIG 2:**
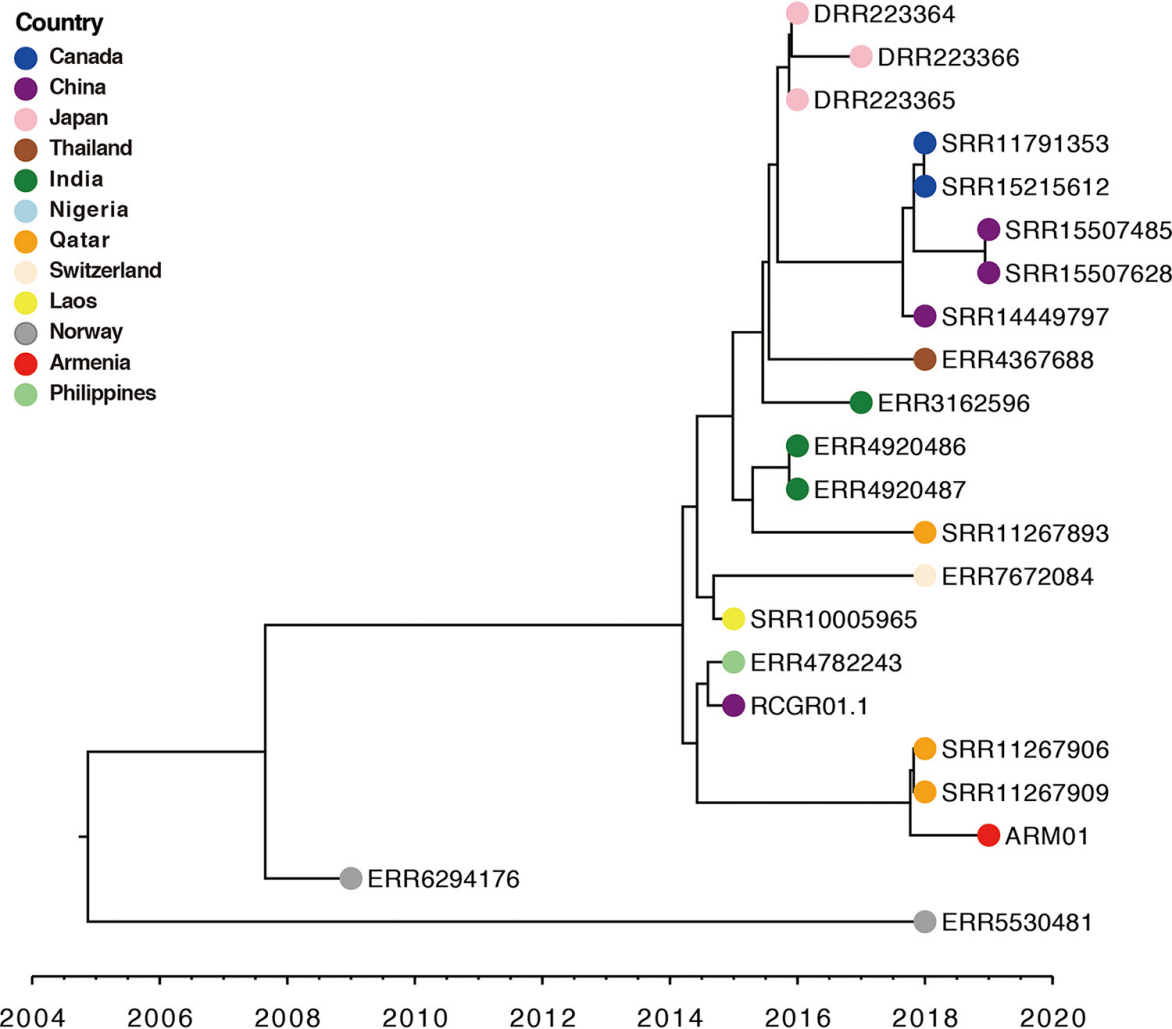
Bayesian evolution analysis of 22 K. pneumoniae ST967 isolates. The color of the circle at the end of each branch represents the country where the isolate was collected.

As to date no whole-genome sequencing studies have been conducted to characterize K. pneumoniae recovered from patients or hospital settings in Armenia, we wanted to estimate the date of the most recent common ancestor (MRCA) of ARM01 and infer its evolutionary origins with those phylogenetically closely related K. pneumoniae isolates. Therefore, we reconstructed a maximum clade credibility (MCC) tree using BEAST (Fig. 2). One isolate obtained from the Pathogenwatch database was removed from the analysis due to the absence of the date of collection. The substitution rate of the K. pneumoniae ST967 maximum likelihood date-calibrated tree was estimated to be 9.59 × 10^−4^ substitutions per site per year (95% confidence interval [CI], 1.73 × 10^−4^ to 2.19 × 10^−3^). The root of the K. pneumoniae ST967 MCC tree constructed was estimated to be approximately 2004 (95% CI, 1998 to 2009), indicating that the K. pneumoniae ST967 is a newly emerged linage that has been recovered in different countries over the last 2 decades (Fig. 2). ARM01 was most closely related to the Qatar isolates (SRR11267906 and SRR11267909) and was found to have a most recent inferred date of divergence from the Qatar isolates, which was 2017 (95% CI, 2017 to 2018). Moreover, pairwise SNP analysis revealed that ARM01 had SNP distances of 27 to SRR11267909 and 45 to SRR11267906 (Table S5). Finally, a spatial phylogeographic reconstruction was performed (Fig. S3), confirming that the Armenian isolate was genetically related to the two isolates recovered from Qatar. The Bayes factor for the transmission from Qatar to Armenia was 0.578, with a posterior probability of 0.122, which may be due to the small sample size.

### Comparative analysis of antimicrobial resistance and virulence genes.

The AMR gene profile of ARM01 was compared to those of the 22 K. pneumoniae ST967 isolates obtained from the Pathogenwatch database and previously published studies (32). In total, 48 AMR genes were identified in the resistome of these isolates. A total of 22 out of 23 K. pneumoniae ST967 isolates possessed genes conferring resistance to three or more classes of antibiotic (Fig. 1; Table S6). Only one of the isolates (ERR5530481), which was recovered from a broiler, harbored one AMR gene (*bla*_SHV-27_ gene). A number of AMR genes, including *strA* (15/23), *strB* (15/23), *qnrS1* (15/23), *mphA* (12/23), *sul1* (15/23), *sul2* (17/23), *tet*(A) (16/23), *bla*_TEM-1D_ (13/23), and *bla*_SHV-27_ (19/23), were detected in most of the K. pneumoniae ST967 isolates, including ARM01, suggesting that multidrug resistance was preserved in the ST967 lineage recovered from a human host. Moreover, we detected 15 genes associated with β-lactam antibiotics in all isolates, including 6 ESBL-associated genes conferring resistance to third-generation cephalosporins (*bla*_CTX-M-3_, *bla*_CTX-M-14_, *bla*_CTX-M-15_, *bla*_SHV-12_, *bla*_SHV-12_, and *bla*_SHV-27_ +178H) and 3 genes conferring resistance to carbapenems (*bla*_NDM-1_, *bla*_KPC-2_, and *bla*_CTX-M-33_). Among the identified ESBL-associated genes, *bla*_CTX-M-15_ and *bla*_CTX-M-3_ were the most common. *bla*_SHV27_ was present in most of the K. pneumoniae ST967 isolates (19/23 [82.6%]). On average, each K. pneumoniae ST967 isolate possessed 12 AMR genes resistant to various classes of antibiotics, which was consistent with the number of AMR genes we found in ARM01 (*n* = 13) (Fig. 1). We also found that ARM01 shared many of the same resistance genes with two Qatar isolates (SRR11267906 and SRR11267909) for β-lactamases (*bla*_SHV-27_), ESBL (*bla*_CTX-M-15_), trimethoprim (*dfrA12*), sulfonamides (*sul1* and *sul2*), phenicol (*catII.2*), macrolides (*mphA*), fluoroquinolones (*qnrS1*), and aminoglycosides [*aadA2* and *aph3(3)-IIa*]. ARM01 also shared aminoglycoside resistance genes (*strA* and *strB*) with one of the Qatar isolates, SSR11267909 (Fig. 1; Fig. S4).

We further compared virulence genes identified in K. pneumoniae ARM01 with those detected in the other 22 K. pneumoniae ST967 isolates (Table S7). Overall, 3 virulence genes were identified. One known virulence factor, *yagZ*/*ecpA*, was detected in all K. pneumoniae ST967 isolates. ERR7672084 (recovered from Switzerland) and ERR4782243 (recovered from Philippines) possessed additional aerobactin-associated genes: *iucC* and *iucB*.

## DISCUSSION

Whole-genome sequencing (WGS) surveillance of K. pneumoniae is imperative due to its increased resistance to antibiotics and high mortality rate (12). Its rapid dissemination as a nosocomial and community-associated pathogen globally has led to public health crises (1, 10). As a tool, WGS can help advance our knowledge and understanding of pathogen global dissemination issues (33). However, WGS surveillance of K. pneumoniae is still scarce in LMICs, leaving essential knowledge gaps in genomic and epidemiological monitoring of this species. To date, only a few studies of K. pneumoniae ST967 have been reported (34, 35), with none from Armenia. To the best of our knowledge, this is the first study reporting on whole-genome sequencing of K. pneumoniae ST967 recovered from a patient in Armenia.

In this study, we report the whole-genome sequencing and comparative analysis of a multidrug-resistant (resistant to 7 out of 11 antibiotics tested) K. pneumoniae ST967 isolate, designated ARM01. WGS analysis revealed that ARM01 carried 13 AMR genes conferring resistance to β-lactamase (*bla*_CTX-M-15_ and *bla*_SHV-27_), trimethoprim (*dfrA12*), tetracycline [*tet*(A)], sulfonamides (*sul1* and *sul2*), phenicols (*catII.2*), macrolides (*mphA*), fluoroquinolones (*qnrS1*), and aminoglycosides (*aadA2*, *aph3-Ia*, *strA*, and *strB*) (Fig. 1; see Table S6 in the supplemental material). Notably, there was a strong correlation between the results of phenotypic antibiotic susceptibility and the antibiotic resistance predicted by the AMR genes. Multidrug resistance was preserved throughout the K. pneumoniae ST967 lineage, with 22 out of 23 isolates harboring multiple AMR genes predicted to be resistant to three or more classes of antibiotics.

Hospital outbreaks of MDR K. pneumoniae, particularly in neonatal units, have often been associated with CTX-M types of ESBL producers (10, 36). Due to the risk of easy dissemination of AMR genes to other bacterial species through mobile genetic elements (MGEs), such as transposons and integrons, ESBLs have become a serious issue (37). In this study, 73.9% (17/23) of K. pneumoniae ST967 isolates were positive for ESBL production, with *bla*_CTX-M-3_ (7/23) and *bla*_CTX-M-15_ (7/23) being the most common ESBL genes detected. A recent global surveillance program that was carried out in 48 countries from 2015 to 2019 showed that the Middle East/Africa had the highest prevalence of ESBL non-carbapenem-resistant *Enterobacteriaceae* (non-CRE) K. pneumoniae isolates, ranging from 32.1% to 39.0%, and that, in Eastern Europe, the rates ranged from 22.0% to 33.1%, with Lithuania having the highest prevalence rate at 55.2% (38).

As a CTX-M-1 group member, *bla*_CTX-M-3_ was originally disseminated in Poland and later (in the 2000s) in Europe (39), and it has only been reported on a few occasions (40, 41). Interestingly, all *bla*_CTX-M-3_ isolates in this study were collected in Asia (Japan, China, and Philippines). Both belong to the CTX-M-1 group: *bla*_CTX-M-15_ differed from *bla*_CTX-M-3_ by a single amino acid change (an asparagine-to-glycine substitution in position ABL238) (42). *bla*_CTX-M-15_ has widely spread around the whole world and is known to hydrolyze many of the cephalosporins. *bla*_CTX-M-15_ was reported to be associated with epidemic MDR K. pneumoniae isolates (ST15, ST147, and ST336) (43). In addition, *bla*_CTX-M-15_ is prevalent in 93.7% (89/95 isolates) of K. pneumoniae ST307 isolates globally (44). *bla*_SHV-27_ was shown to be frequently associated with CTX-M-type ESBLs (45). *bla*_SHV-27_ was commonly found in K. pneumoniae, Escherichia coli, and Enterobacter cloacae isolates recovered from human clinical samples and companion animals (46–48). In this study, *bla*_SHV-27_ was detected both in ARM01 as well as in K. pneumoniae isolates belonging to the ST967 lineage (19/23 isolates). Moreover, ARM01 displayed nearly identical AMR and plasmid profiles to one of the Qatar isolates (SRR11267909), suggesting their close genetic relatedness.

In contrast to the hypervirulent K. pneumoniae isolates, ARM01 did not carry genes for any of the classical virulence factors, such as yersiniabactin (*ybt*), colibactin (*clb*), salmochelin (*iro*), aerobactin (*iuc*), or hypermucoidy (*rmpA* and *rmpA2*) (49, 50). These virulence-associated genes have been associated with invasive infections and were frequently found in hypervirulent K. pneumoniae isolates (51). The absence of some typical virulence determinants (such as *rmpA*, *rmpA2*, *iuc*, and *iro*) has previously been shown to result in nonhypervirulent phenotypes (44, 52), indicating the relatively low pathogenicity of ARM01. None of the K. pneumoniae ST967 isolates, with the exception of ERR4782243 and ERR7672084, contained the canonical hypervirulence markers; however, all K. pneumoniae ST967 isolates acquired the *yagZ*/*ecpA* gene. As a gene coding for an E. coli common pilus adherence factor, *yagZ*/*ecpA* has been reported to be involved in cell adhesion and biofilm formation (53). It was commonly found in K. pneumoniae isolates belonging to various sequence types previously recovered from patients (54). Similar to this, another study showed that 96% (66/69) of the hospital-acquired K. pneumoniae isolates harbored *yagZ*/*ecpA* and that there was a 94% phenotypic correlation of ECP production, which was essential for the formation of the adhesive structure (55).

Time-calibrated phylogenetic analysis estimated the MRCA of K. pneumoniae ST967 lineage to be 2004, implying that the K. pneumoniae ST967 subgroup was a newly emerged clone that had spread across countries over the last 2 decades. Comparative analysis revealed that ARM01 was genetically similar to two Qatar isolates (SRR11267906 and SRR11267909). SRR11267906 and SRR11267909 represent case sequencing data recovered from the same hospital (35) with 64 SNP differences, whereas the numbers of SNP differences between ARM01 and SRR11267909 and SRR11267906 were 27 and 45, respectively. It has previously been reported that two bacterial isolates may be considered closely related clones if they possess a relatively low SNP distance (fewer than 50 SNPs) due to the short divergence time (56, 57). S. David et al. (58) showed that the most frequent minimum SNP distance for carbapenem-resistant K. pneumoniae ST258/512 (found in 32 different European countries) was 45 SNPs and was considered an international spread. Using spatial phylogenetic reconstruction of K. pneumoniae ST967, we reconstructed its transmission dynamics between countries, which indeed shed light on the high genetic relatedness of ARM01 and the Qatar isolates (SRR11267906 and SRR11267909). Undoubtably, there is an urgent need for additional data collection and analysis of this newly emerged K. pneumoniae lineage in Armenia, but also further molecular epidemiology investigations are warranted to reveal its evolutionary origins.

### Conclusions.

This study revealed the genomic characteristics of ARM01, an isolate belonging to the newly emerged K. pneumoniae ST967 lineage. Multilevel analysis found it to be genetically highly similar to two Qatar isolates, which shared and had descended from a common ancestor in 2017. ARM01 was resistant to a wide range of antibiotics despite harboring just one known virulence-associated gene. Additional surveillance studies are warranted to further understand the prevalence and molecular epidemiology of K. pneumoniae and other Gram-negative pathogens circulating in health care and community settings in Armenia and to identify possible risk factors, antimicrobial resistance associated with these isolates, genetic diversity, and disease burden in this country to inform National Infection Prevention and Control Policy.

Although our data demonstrate compelling evidence of characteristics of ARM01 and phylogeny of the K. pneumoniae ST967 lineage, there are several limitations to our study. The main limitation of this study is the small sample size both received for this study and those publicly available data. The fact that only one out of the eight K. pneumoniae isolates received for this study was ST967 is also a major limitation as we could not fully decipher the population structure of K. pneumoniae ST967 in Armenia. Furthermore, only 22 K. pneumoniae ST967 data were publicly available, and therefore, comparative genomics analysis of this recently emerged subtype remains to be explored. In addition, epidemiological data were lacking; we do not know if there were any associated risk factors, i.e., travel abroad.

## MATERIALS AND METHODS

### Identification and antibiotic susceptibility testing.

Eight K. pneumoniae isolates were received from the Medical Microbiology laboratories of three hospitals in Armenia between January 2019 and August 2019. All isolates were recovered from various clinical specimens (urine, sputum, throat, blood, and stool) of hospitalized patients. The isolates were identified using a matrix-assisted laser desorption ionization–time of flight mass spectrometry (MALDI-TOF-MS) as described previously (59).

All isolates were tested using a disk diffusion method for susceptibility to a panel of 11 antibiotics, including ampicillin (10 mg), piperacillin-tazobactam (30/6 mg), amoxicillin-clavulanic acid (20 and 10 mg, respectively), ceftazidime (10 mg), cefepime (30 mg), norfloxacin (10 mg), levofloxacin (5 mg), amikacin (30 mg), imipenem (10 mg), meropenem (10 mg), and chloramphenicol (30 mg) (Mast Group, Merseyside, United Kingdom) according to the European Committee on Antimicrobial Susceptibility Testing protocol (EUCAST v.6.0, 2017) (60). The antibiotics chosen were those most frequently used in clinical settings in Armenia. Isolates resistant to three or more antibiotic classes were considered multidrug resistant.

### Whole-genome sequencing and assembly.

Genomic DNA was extracted using the TIANamp bacterial DNA kit (Tiangen, China), and paired-end sequencing libraries were constructed using Nextera XT DNA sample preparation kits or the TruSeq DNA HT sample prep kit (Illumina, USA) following the manufacturer’s instructions. Whole-genome sequencing was performed using the Illumina HiSeq platform, with a minimum coverage of 100-fold and generating 151-bp sequence reads. The quality of raw reads was assessed using FASTQC v.0.11.9 (https://github.com/s-andrews/FastQC) and trimmed using Trimmomatic v.0.38 to eliminate adaptors and low-quality sequences (61). Trimmed reads were *de novo* assembled using Velvet v.1.2.10 (62), with contigs less than 500 bp being removed. Prokka v.1.14.5 was used for draft genome annotation (63).

### Multilocus sequence typing and bioinformatic analysis.

Multilocus sequence typing (MLST) was conducted using mlst v.2.19.0 (https://github.com/tseemann/mlst) (the most updated version from pubMLST, accessed November 2021) (64). The sequence type for each isolate was determined by seven housekeeping genes: *gapA*, *infB*, *mdh*, *pgi*, *phoE*, *rpoB*, and *tonB* (65). Kleborate v.0.3.0 (66) was used for K (capsule) and O antigen (LPS) serotype prediction via *wzi* alleles and Kaptive (67). Kleborate was also used to identify AMR genes, with a BLAST cutoff set at 90% identity and 80% coverage. Abricate v.1.0.1 (https://github.com/tseemann/abricate) was used to identify virulence determinants using the VFDB database (accessed November 2021) (68). Plasmid prediction was performed by searching for plasmid replicons against the PlasmidFinder database (accessed November 2021) (69) using Staramr v.0.7.2 (https://github.com/phac-nml/staramr). The Python module Pyani v.0.2.11 was used to calculate the pairwise average nucleotide identity (ANI) between all whole-genome-sequenced isolates (70). In addition, snp-dists (https://github.com/tseemann/snp-dists) was used to convert a FASTA alignment to an SNP distance matrix.

### Phylogenetic analyses.

Phylogenetic analyses were conducted to compare ARM01 with 22 previously reported K. pneumoniae ST967 genomes obtained from the Pathogenwatch database (https://pathogen.watch) (accessed October 2021) (71) and previously published studies (34) using Snippy v.4.6.0 (https://github.com/tseemann/snippy). The contigs of all genomes were aligned against a reference K. pneumoniae ST967 genome (GenBank accession no. AP023148.1) (34), and recombination was filtered by Gubbins v.2.4.1 (72). A maximum likelihood phylogenetic SNP tree was constructed from the aligned sequences using FastTree v.2.1.11 (73) based on the GTR+GAMMA model, with 100 bootstrap (BS) replications. The phylogenetic tree was visualized and annotated in Evolview (74, 75).

### Temporal and spatial phylogenetic reconstruction.

To reconstruct the time-calibrated phylogeny of all isolates, we conducted a Bayesian analysis using BEAST v.1.8.4 package (76). jModelTest v.2.1.10 (77) was used to determine the best-fit model and parameters of nucleotide substitution. A coalescent Bayesian skyline model for population growth was performed, with a relaxed clock and a general time-reversible (GTR) nucleotide substitution model. BEAST was run across four independent iterations, each set to a Markov chain Monte Carlo (MCMC) of 10 million generations with samples taken at every 1,000 generations. LogCombiner was used to combine output files from four independent runs. A combined log file was analyzed with Tracer v.1.7.2. The merged tree file was used to build the maximum clade credibility (MCC) tree, which was then displayed by FigTree v.1.4.4. The Bayesian stochastic search variable selection (BSSVS) method was used to establish a Bayesian factor (BF) test to infer the possibility of spatial geographic transmission based on discrete geographic information. The MCC tree with phylogeographic reconstruction was generated with 10% burn-in using the Tree Annotator. SpreaD v1.0.7 (78) was used to visualize the location-annotated MCC tree.

### Pan-genome analysis.

The annotated genome assemblies were used to construct the pan-genome analysis using Roary v.3.13.0 (79). Accessory genes were classified as genes found in less than 99% of all isolates. To compare the accessory gene profiles among all isolates, a hierarchically clustered heat map was constructed using the Pearson product-moment correlation coefficient in R v.3.6.2 (R Core Team; https://www.R-project.org) according to the presence (1) and absence (0) of all accessory genes.

### Data availability.

The short-read sequencing data generated in this study were deposited in the ENA database under the accession no. ERR9882335. Individual accession numbers for the genome sequencing data are included in Table S2.

## References

[1] Cardoso B, Esposito F, Fontana H, Fuga B, Moura Q, Sano E, Sato MIZ, Brandão CJ, Oliveira FA, Levy CE, Lincopan N. 2022. Genomic analysis of a Kpi (pilus system)-positive and CTX-M-15-producing Klebsiella pneumoniae belonging to the high-risk clone ST15 isolated from an impacted river in Brazil. Genomics 114:378–383. doi:10.1016/j.ygeno.2021.12.007. 34923088

[2] Singh RP, Jha P, Jha PN. 2015. The plant-growth-promoting bacterium Klebsiella sp. SBP-8 confers induced systemic tolerance in wheat (Triticum aestivum) under salt stress. J Plant Physiol 184:57–67. doi:10.1016/j.jplph.2015.07.002. 26217911

[3] Sellera FP, Lopes R, Monte DFM, Cardoso B, Esposito F, Anjos CD, da Silva L, Lincopan N. 2020. Genomic analysis of multidrug-resistant CTX-M-15-positive Klebsiella pneumoniae belonging to the highly successful ST15 clone isolated from a dog with chronic otitis. J Glob Antimicrob Resist 22:659–661. doi:10.1016/j.jgar.2020.06.017. 32619688

[4] Saidani M, Messadi L, Mefteh J, Chaouechi A, Soudani A, Selmi R, Dâaloul-Jedidi M, Ben Chehida F, Mamlouk A, Jemli MH, Madec JY, Haenni M. 2019. Various Inc-type plasmids and lineages of Escherichia coli and Klebsiella pneumoniae spreading bla_CTX-M-15_, bla_CTX-M-1_ and mcr-1 genes in camels in Tunisia. J Glob Antimicrob Resist 19:280–283. doi:10.1016/j.jgar.2019.05.007. 31100503

[5] Founou RC, Founou LL, Allam M, Ismail A, Essack SY. 2019. Whole genome sequencing of extended spectrum β-lactamase (ESBL)-producing Klebsiella pneumoniae isolated from hospitalized patients in KwaZulu-Natal, South Africa. Sci Rep 9:6266. doi:10.1038/s41598-019-42672-2. 31000772 PMC6472517

[6] Marques C, Menezes J, Belas A, Aboim C, Cavaco-Silva P, Trigueiro G, Telo Gama L, Pomba C. 2019. Klebsiella pneumoniae causing urinary tract infections in companion animals and humans: population structure, antimicrobial resistance and virulence genes. J Antimicrob Chemother 74:594–602. doi:10.1093/jac/dky499. 30535393

[7] Tan TY, Ong M, Cheng Y, Ng LSY. 2019. Hypermucoviscosity, rmpA, and aerobactin are associated with community-acquired Klebsiella pneumoniae bacteremic isolates causing liver abscess in Singapore. J Microbiol Immunol Infect 52:30–34. doi:10.1016/j.jmii.2017.07.003. 28736222

[8] Li B, Li M, Qu L, Wang M, Guo J. 2014. Prevalence and characteristics of extended-spectrum beta-lactamase-producing Klebsiella pneumoniae isolated from pediatric inpatients with respiratory tract infections at a teaching hospital in China. Scand J Infect Dis 46:200–203. doi:10.3109/00365548.2013.859393. 24359516

[9] Stebbings AE, Ti TY, Tan WC. 1999. Hospital acquired pneumonia in the medical intensive care unit—a prospective study. Singapore Med J 40:508–512. 10572489

[10] Podschun R, Ullmann U. 1998. Klebsiella spp. as nosocomial pathogens: epidemiology, taxonomy, typing methods, and pathogenicity factors. Clin Microbiol Rev 11:589–603. doi:10.1128/CMR.11.4.589. 9767057 PMC88898

[11] WHO Regional Office for Europe, European Centre for Disease Prevention and Control. 2021. Surveillance of antimicrobial resistance in Europe, 2020 data. Executive summary. https://www.ecdc.europa.eu/en/publications-data/surveillance-antimicrobial-resistance-europe-2020.

[12] Xu L, Sun X, Ma X. 2017. Systematic review and meta-analysis of mortality of patients infected with carbapenem-resistant Klebsiella pneumoniae. Ann Clin Microbiol Antimicrob 16:18. doi:10.1186/s12941-017-0191-3. 28356109 PMC5371217

[13] Wu D, Ding J, Jia Y, Liu H, Xiao J, Peng J. 2021. Predictors of mortality in acute pancreatitis complicated with multidrug-resistant Klebsiella pneumoniae infection. BMC Infect Dis 21:977. doi:10.1186/s12879-021-06709-0. 34544384 PMC8451102

[14] Peirano G, Chen L, Kreiswirth BN, Pitout JDD. 2020. Emerging antimicrobial-resistant high-risk Klebsiella pneumoniae clones ST307 and ST147. Antimicrob Agents Chemother 64:e01148-20. doi:10.1128/AAC.01148-20. 32747358 PMC7508593

[15] Roe CC, Vazquez AJ, Esposito EP, Zarrilli R, Sahl JW. 2019. Diversity, virulence, and antimicrobial resistance in isolates from the newly emerging Klebsiella pneumoniae ST101 lineage. Front Microbiol 10:542. doi:10.3389/fmicb.2019.00542. 31001209 PMC6454207

[16] Diallo OO, Baron SA, Abat C, Colson P, Chaudet H, Rolain JM. 2020. Antibiotic resistance surveillance systems: a review. J Glob Antimicrob Resist 23:430–438. doi:10.1016/j.jgar.2020.10.009. 33176216

[17] Chong Y, Yakushiji H, Ito Y, Kamimura T. 2011. Clinical and molecular epidemiology of extended-spectrum β-lactamase-producing Escherichia coli and Klebsiella pneumoniae in a long-term study from Japan. Eur J Clin Microbiol Infect Dis 30:83–87. doi:10.1007/s10096-010-1057-1. 20859753

[18] Calbo E, Garau J. 2015. The changing epidemiology of hospital outbreaks due to ESBL-producing Klebsiella pneumoniae: the CTX-M-15 type consolidation. Future Microbiol 10:1063–1075. doi:10.2217/fmb.15.22. 26059626

[19] Mshana SE, Hain T, Domann E, Lyamuya EF, Chakraborty T, Imirzalioglu C. 2013. Predominance of Klebsiella pneumoniae ST14 carrying CTX-M-15 causing neonatal sepsis in Tanzania. BMC Infect Dis 13:466. doi:10.1186/1471-2334-13-466. 24099282 PMC3851032

[20] Sartori L, Sellera FP, Moura Q, Cardoso B, Cerdeira L, Lincopan N. 2019. Multidrug-resistant CTX-M-15-positive Klebsiella pneumoniae ST307 causing urinary tract infection in a dog in Brazil. J Glob Antimicrob Resist 19:96–97. doi:10.1016/j.jgar.2019.09.003. 31520809

[21] Davies J, Davies D. 2010. Origins and evolution of antibiotic resistance. Microbiol Mol Biol Rev 74:417–433. doi:10.1128/MMBR.00016-10. 20805405 PMC2937522

[22] Cox JA, Vlieghe E, Mendelson M, Wertheim H, Ndegwa L, Villegas MV, Gould I, Levy Hara G. 2017. Antibiotic stewardship in low- and middle-income countries: the same but different? Clin Microbiol Infect 23:812–818. doi:10.1016/j.cmi.2017.07.010. 28712667

[23] Okeke IN, Laxminarayan R, Bhutta ZA, Duse AG, Jenkins P, O'Brien TF, Pablos-Mendez A, Klugman KP. 2005. Antimicrobial resistance in developing countries. Part I. Recent trends and current status. Lancet Infect Dis 5:481–493. doi:10.1016/S1473-3099(05)70189-4. 16048717

[24] Lepuschitz S, Schill S, Stoeger A, Pekard-Amenitsch S, Huhulescu S, Inreiter N, Hartl R, Kerschner H, Sorschag S, Springer B, Brisse S, Allerberger F, Mach RL, Ruppitsch W. 2019. Whole genome sequencing reveals resemblance between ESBL-producing and carbapenem resistant Klebsiella pneumoniae isolates from Austrian rivers and clinical isolates from hospitals. Sci Total Environ 662:227–235. doi:10.1016/j.scitotenv.2019.01.179. 30690357

[25] Wang G, Zhao G, Chao X, Xie L, Wang H. 2020. The characteristic of virulence, biofilm and antibiotic resistance of Klebsiella pneumoniae. Int J Environ Res Public Health 17:6278. doi:10.3390/ijerph17176278. 32872324 PMC7503635

[26] Rimoldi SG, Gentile B, Pagani C, Di Gregorio A, Anselmo A, Palozzi AM, Fortunato A, Pittiglio V, Ridolfo AL, Gismondo MR, Rizzardini G, Lista F. 2017. Whole genome sequencing for the molecular characterization of carbapenem-resistant Klebsiella pneumoniae strains isolated at the Italian ASST Fatebenefratelli Sacco Hospital, 2012–2014. BMC Infect Dis 17:666. doi:10.1186/s12879-017-2760-7. 29017452 PMC5634883

[27] Meletis G, Chatzopoulou F, Chatzidimitriou D, Tsingerlioti F, Botziori C, Tzimagiorgis G, Skoura L. 2019. Whole genome sequencing of NDM-1-producing ST11 Klebsiella pneumoniae isolated in a private laboratory in Greece. Microb Drug Resist 25:80–86. doi:10.1089/mdr.2017.0411. 29698126

[28] Iskandar K, Molinier L, Hallit S, Sartelli M, Hardcastle TC, Haque M, Lugova H, Dhingra S, Sharma P, Islam S, Mohammed I, Naina Mohamed I, Hanna PA, Hajj SE, Jamaluddin NAH, Salameh P, Roques C. 2021. Surveillance of antimicrobial resistance in low- and middle-income countries: a scattered picture. Antimicrob Resist Infect Control 10:63. doi:10.1186/s13756-021-00931-w. 33789754 PMC8011122

[29] Davtyan H, Grigoryan R, Niazyan L, Davidyants M, Ghalechyan T, Davtyan K. 2021. Antimicrobial resistance in a tertiary care hospital in Armenia: 2016-2019. Trop Med Infect Dis 6:31. doi:10.3390/tropicalmed6010031. 33800026 PMC8005984

[30] Mkrtchyan HV, Xu Z, Yacoub M, Ter-Stepanyan MM, Karapetyan HD, Kearns AM, Cutler RR, Pichon B, Hambardzumyan AD. 2017. Detection of diverse genotypes of methicillin-resistant Staphylococcus aureus from hospital personnel and the environment in Armenia. Antimicrob Resist Infect Control 6:19. doi:10.1186/s13756-017-0169-0. 28184301 PMC5294836

[31] Jamrozy D, Misra R, Xu Z, Ter-Stepanyan MM, Kocharyan KS, Cave R, Hambardzumyan AD, Mkrtchyan HV. 2019. Novel methicillin-resistant Staphylococcus aureus CC8 clone identified in a hospital setting in Armenia. Front Microbiol 10:1592. doi:10.3389/fmicb.2019.01592. 31354680 PMC6635598

[32] Chi X, Berglund B, Zou H, Zheng B, Börjesson S, Ji X, Ottoson J, Lundborg CS, Li X, Nilsson LE. 2019. Characterization of clinically relevant strains of extended-spectrum β-lactamase-producing Klebsiella pneumoniae occurring in environmental sources in a rural area of China by using whole-genome sequencing. Front Microbiol 10:211. doi:10.3389/fmicb.2019.00211. 30809212 PMC6379450

[33] Kwong JC, McCallum N, Sintchenko V, Howden BP. 2015. Whole genome sequencing in clinical and public health microbiology. Pathology 47:199–210. doi:10.1097/PAT.0000000000000235. 25730631 PMC4389090

[34] Sato T, Wada T, Nishijima S, Fukushima Y, Nakajima C, Suzuki Y, Takahashi S, Yokota SI. 2020. Emergence of the novel aminoglycoside acetyltransferase variant aac(6′)-Ib-D179Y and acquisition of colistin heteroresistance in carbapenem-resistant Klebsiella pneumoniae due to a disrupting mutation in the DNA repair enzyme MutS. mBio 11:e01954-20. doi:10.1128/mBio.01954-20. 33443109 PMC8534291

[35] Perez-Lopez A, Sundararaju S, Al-Mana H, Tsui KM, Hasan MR, Suleiman M, Janahi M, Al Maslamani E, Tang P. 2020. Molecular characterization of extended-spectrum β-lactamase-producing Escherichia coli and Klebsiella pneumoniae among the pediatric population in Qatar. Front Microbiol 11:581711. doi:10.3389/fmicb.2020.581711. 33262745 PMC7686840

[36] Chong Y, Shimoda S, Shimono N. 2018. Current epidemiology, genetic evolution and clinical impact of extended-spectrum β-lactamase-producing Escherichia coli and Klebsiella pneumoniae. Infect Genet Evol 61:185–188. doi:10.1016/j.meegid.2018.04.005. 29626676

[37] Paterson DL, Bonomo RA. 2005. Extended-spectrum beta-lactamases: a clinical update. Clin Microbiol Rev 18:657–686. doi:10.1128/CMR.18.4.657-686.2005. 16223952 PMC1265908

[38] Karlowsky JA, Lob SH, DeRyke CA, Siddiqui F, Young K, Motyl MR, Sahm DF. 2022. Prevalence of ESBL non-CRE Escherichia coli and Klebsiella pneumoniae among clinical isolates collected by the SMART global surveillance programme from 2015 to 2019. Int J Antimicrob Agents 59:106535. doi:10.1016/j.ijantimicag.2022.106535. 35091052

[39] Livermore DM, Canton R, Gniadkowski M, Nordmann P, Rossolini GM, Arlet G, Ayala J, Coque TM, Kern-Zdanowicz I, Luzzaro F, Poirel L, Woodford N. 2006. CTX-M: changing the face of ESBLs in Europe. J Antimicrob Chemother 59:165–174. doi:10.1093/jac/dkl483. 17158117

[40] Bevan ER, Jones AM, Hawkey PM. 2017. Global epidemiology of CTX-M β-lactamases: temporal and geographical shifts in genotype. J Antimicrob Chemother 72:2145–2155. doi:10.1093/jac/dkx146. 28541467

[41] Furlan JPR, Lopes R, Gonzalez IHL, Ramos PL, von Zeska Kress MR, Stehling EG. 2020. Hypermucoviscous/hypervirulent and extensively drug-resistant QnrB2-, QnrS1-, and CTX-M-3-coproducing Klebsiella pneumoniae ST2121 isolated from an infected elephant (Loxodonta africana). Vet Microbiol 251:108909. doi:10.1016/j.vetmic.2020.108909. 33176213

[42] Karim A, Poirel L, Nagarajan S, Nordmann P. 2001. Plasmid-mediated extended-spectrum beta-lactamase (CTX-M-3 like) from India and gene association with insertion sequence ISEcp1. FEMS Microbiol Lett 201:237–241. doi:10.1111/j.1574-6968.2001.tb10762.x. 11470367

[43] Rodrigues C, Machado E, Ramos H, Peixe L, Novais Â. 2014. Expansion of ESBL-producing Klebsiella pneumoniae in hospitalized patients: a successful story of international clones (ST15, ST147, ST336) and epidemic plasmids (IncR, IncFIIK). Int J Med Microbiol 304:1100–1108. doi:10.1016/j.ijmm.2014.08.003. 25190354

[44] Wyres KL, Hawkey J, Hetland MAK, Fostervold A, Wick RR, Judd LM, Hamidian M, Howden BP, Löhr IH, Holt KE. 2019. Emergence and rapid global dissemination of CTX-M-15-associated Klebsiella pneumoniae strain ST307. J Antimicrob Chemother 74:577–581. doi:10.1093/jac/dky492. 30517666 PMC6376852

[45] Mondal AH, Siddiqui MT, Sultan I, Haq QMR. 2019. Prevalence and diversity of blaTEM, blaSHV and blaCTX-M variants among multidrug resistant Klebsiella spp. from an urban riverine environment in India. Int J Environ Health Res 29:117–129. doi:10.1080/09603123.2018.1515425. 30185065

[46] Liakopoulos A, Mevius D, Ceccarelli D. 2016. A review of SHV extended-spectrum β-lactamases: neglected yet ubiquitous. Front Microbiol 7:1374. doi:10.3389/fmicb.2016.01374. 27656166 PMC5011133

[47] Zou LK, Wang HN, Zeng B, Zhang AY, Li JN, Li XT, Tian GB, Wei K, Zhou YS, Xu CW, Yang ZR. 2011. Phenotypic and genotypic characterization of β-lactam resistance in Klebsiella pneumoniae isolated from swine. Vet Microbiol 149:139–146. doi:10.1016/j.vetmic.2010.09.030. 21035968

[48] Harada K, Shimizu T, Mukai Y, Kuwajima K, Sato T, Usui M, Tamura Y, Kimura Y, Miyamoto T, Tsuyuki Y, Ohki A, Kataoka Y. 2016. Phenotypic and molecular characterization of antimicrobial resistance in Klebsiella spp. isolates from companion animals in Japan: clonal dissemination of multidrug-resistant extended-spectrum β-lactamase-producing Klebsiella pneumoniae. Front Microbiol 7:1021. doi:10.3389/fmicb.2016.01021. 27446056 PMC4925667

[49] Kakuta N, Nakano R, Nakano A, Suzuki Y, Masui T, Horiuchi S, Kakuta R, Tsubaki K, Ogawa M, Yano H. 2020. Molecular characteristics of extended-spectrum β-lactamase-producing Klebsiella pneumoniae in Japan: predominance of CTX-M-15 and emergence of hypervirulent clones. Int J Infect Dis 98:281–286. doi:10.1016/j.ijid.2020.06.083. 32619765

[50] Li W, Sun G, Yu Y, Li N, Chen M, Jin R, Jiao Y, Wu H. 2014. Increasing occurrence of antimicrobial-resistant hypervirulent (hypermucoviscous) Klebsiella pneumoniae isolates in China. Clin Infect Dis 58:225–232. doi:10.1093/cid/cit675. 24099919

[51] Lam MMC, Wyres KL, Judd LM, Wick RR, Jenney A, Brisse S, Holt KE. 2018. Tracking key virulence loci encoding aerobactin and salmochelin siderophore synthesis in Klebsiella pneumoniae. Genome Med 10:77. doi:10.1186/s13073-018-0587-5. 30371343 PMC6205773

[52] Wysocka M, Zamudio R, Oggioni MR, Gołębiewska J, Dudziak A, Krawczyk B. 2020. The new Klebsiella pneumoniae ST152 variants with hypermucoviscous phenotype isolated from renal transplant recipients with asymptomatic bacteriuria—genetic characteristics by WGS. Genes 11:1189. doi:10.3390/genes11101189. 33066176 PMC7601988

[53] Rendón MA, Saldaña Z, Erdem AL, Monteiro-Neto V, Vázquez A, Kaper JB, Puente JL, Girón JA. 2007. Commensal and pathogenic Escherichia coli use a common pilus adherence factor for epithelial cell colonization. Proc Natl Acad Sci USA 104:10637–10642. doi:10.1073/pnas.0704104104. 17563352 PMC1890562

[54] Devanga Ragupathi NK, Muthuirulandi Sethuvel DP, Triplicane Dwarakanathan H, Murugan D, Umashankar Y, Monk PN, Karunakaran E, Veeraraghavan B. 2020. The influence of biofilms on carbapenem susceptibility and patient outcome in device associated K. pneumoniae infections: insights into phenotype vs genome-wide analysis and correlation. Front Microbiol 11:591679. doi:10.3389/fmicb.2020.591679. 33381089 PMC7767932

[55] Alcántar-Curiel MD, Blackburn D, Saldaña Z, Gayosso-Vázquez C, Iovine NM, De la Cruz MA, Girón JA. 2013. Multi-functional analysis of Klebsiella pneumoniae fimbrial types in adherence and biofilm formation. Virulence 4:129–138. doi:10.4161/viru.22974. 23302788 PMC3654611

[56] Emeraud C, Figueiredo S, Bonnin RA, Khecharem M, Ouzani S, Leblanc PE, Jousset AB, Fortineau N, Duranteau J, Dortet L. 2021. Outbreak of CTX-M-15 extended-spectrum β-lactamase-producing Klebsiella pneumoniae ST394 in a French intensive care unit dedicated to COVID-19. Pathogens 10:1426. doi:10.3390/pathogens10111426. 34832582 PMC8618658

[57] Pang R, Wu S, Zhang F, Huang J, Wu H, Zhang J, Li Y, Ding Y, Zhang J, Chen M, Wei X, Zhang Y, Gu Q, Zhou Z, Liang B, Li W, Wu Q. 2020. The genomic context for the evolution and transmission of community-associated Staphylococcus aureus ST59 through the food chain. Front Microbiol 11:422. doi:10.3389/fmicb.2020.00422. 32256477 PMC7090029

[58] David S, Reuter S, Harris SR, Glasner C, Feltwell T, Argimon S, Abudahab K, Goater R, Giani T, Errico G, Aspbury M, Sjunnebo S, EuSCAPE Working Group, ESGEM Study Group, Feil EJ, Rossolini GM, Aanensen DM, Grundmann H. 2019. Epidemic of carbapenem-resistant Klebsiella pneumoniae in Europe is driven by nosocomial spread. Nat Microbiol 4:1919–1929. doi:10.1038/s41564-019-0492-8. 31358985 PMC7244338

[59] Mkrtchyan HV, Russell CA, Wang N, Cutler RR. 2013. Could public restrooms be an environment for bacterial resistomes? PLoS One 8:e54223. doi:10.1371/journal.pone.0054223. 23349833 PMC3547874

[60] European Committee on Antimicrobial Susceptibility Testing. 2017. Breakpoint tables for interpretation of MICs and zone diameters. Version 7.1. http://www.eucast.org.

[61] Bolger AM, Lohse M, Usadel B. 2014. Trimmomatic: a flexible trimmer for Illumina sequence data. Bioinformatics 30:2114–2120. doi:10.1093/bioinformatics/btu170. 24695404 PMC4103590

[62] Zerbino DR, Birney E. 2008. Velvet: algorithms for de novo short read assembly using de Bruijn graphs. Genome Res 18:821–829. doi:10.1101/gr.074492.107. 18349386 PMC2336801

[63] Seemann T. 2014. Prokka: rapid prokaryotic genome annotation. Bioinformatics 30:2068–2069. doi:10.1093/bioinformatics/btu153. 24642063

[64] Jolley KA, Bray JE, Maiden MCJ. 2018. Open-access bacterial population genomics: BIGSdb software, the PubMLST.org website and their applications. Wellcome Open Res 3:124. doi:10.12688/wellcomeopenres.14826.1. 30345391 PMC6192448

[65] Diancourt L, Passet V, Verhoef J, Grimont PA, Brisse S. 2005. Multilocus sequence typing of Klebsiella pneumoniae nosocomial isolates. J Clin Microbiol 43:4178–4182. doi:10.1128/JCM.43.8.4178-4182.2005. 16081970 PMC1233940

[66] Lam MMC, Wick RR, Watts SC, Cerdeira LT, Wyres KL, Holt KE. 2021. A genomic surveillance framework and genotyping tool for Klebsiella pneumoniae and its related species complex. Nat Commun 12:4188. doi:10.1038/s41467-021-24448-3. 34234121 PMC8263825

[67] Wyres KL, Wick RR, Gorrie C, Jenney A, Follador R, Thomson NR, Holt KE. 2016. Identification of Klebsiella capsule synthesis loci from whole genome data. Microb Genom 2:e000102. doi:10.1099/mgen.0.000102. 28348840 PMC5359410

[68] Liu B, Zheng D, Zhou S, Chen L, Yang J. 2022. VFDB 2022: a general classification scheme for bacterial virulence factors. Nucleic Acids Res 50:D912–D917. doi:10.1093/nar/gkab1107. 34850947 PMC8728188

[69] Carattoli A, Zankari E, García-Fernández A, Voldby Larsen M, Lund O, Villa L, Møller Aarestrup F, Hasman H. 2014. In silico detection and typing of plasmids using PlasmidFinder and plasmid multilocus sequence typing. Antimicrob Agents Chemother 58:3895–3903. doi:10.1128/AAC.02412-14. 24777092 PMC4068535

[70] Pritchard L, Glover RH, Humphris S, Elphinstone JG, Toth IK. 2016. Genomics and taxonomy in diagnostics for food security: soft-rotting enterobacterial plant pathogens. Anal Methods 8:12–24. doi:10.1039/C5AY02550H.

[71] Argimón S, David S, Underwood A, Abrudan M, Wheeler NE, Kekre M, Abudahab K, Yeats CA, Goater R, Taylor B, Harste H, Muddyman D, Feil EJ, Brisse S, Holt K, Donado-Godoy P, Ravikumar KL, Okeke IN, Carlos C, Aanensen DM, NIHR Global Health Research Unit on Genomic Surveillance of Antimicrobial Resistance. 2021. Rapid genomic characterization and global surveillance of Klebsiella using Pathogenwatch. Clin Infect Dis 73:S325–S335. doi:10.1093/cid/ciab784. 34850838 PMC8634497

[72] Croucher NJ, Page AJ, Connor TR, Delaney AJ, Keane JA, Bentley SD, Parkhill J, Harris SR. 2015. Rapid phylogenetic analysis of large samples of recombinant bacterial whole genome sequences using Gubbins. Nucleic Acids Res 43:e15. doi:10.1093/nar/gku1196. 25414349 PMC4330336

[73] Price MN, Dehal PS, Arkin AP. 2010. FastTree 2—approximately maximum-likelihood trees for large alignments. PLoS One 5:e9490. doi:10.1371/journal.pone.0009490. 20224823 PMC2835736

[74] Zhang H, Gao S, Lercher MJ, Hu S, Chen WH. 2012. EvolView, an online tool for visualizing, annotating and managing phylogenetic trees. Nucleic Acids Res 40:W569–W572. doi:10.1093/nar/gks576. 22695796 PMC3394307

[75] He Z, Zhang H, Gao S, Lercher MJ, Chen WH, Hu S. 2016. Evolview v2: an online visualization and management tool for customized and annotated phylogenetic trees. Nucleic Acids Res 44:W236–W241. doi:10.1093/nar/gkw370. 27131786 PMC4987921

[76] Drummond AJ, Suchard MA, Xie D, Rambaut A. 2012. Bayesian phylogenetics with BEAUti and the BEAST 1.7. Mol Biol Evol 29:1969–1973. doi:10.1093/molbev/mss075. 22367748 PMC3408070

[77] Darriba D, Taboada GL, Doallo R, Posada D. 2012. jModelTest 2: more models, new heuristics and parallel computing. Nat Methods 9:772. doi:10.1038/nmeth.2109. PMC4594756 22847109

[78] Bielejec F, Rambaut A, Suchard MA, Lemey P. 2011. SPREAD: Spatial Phylogenetic Reconstruction of Evolutionary Dynamics. Bioinformatics 27:2910–2912. doi:10.1093/bioinformatics/btr481. 21911333 PMC3187652

[79] Page AJ, Cummins CA, Hunt M, Wong VK, Reuter S, Holden MT, Fookes M, Falush D, Keane JA, Parkhill J. 2015. Roary: rapid large-scale prokaryote pan genome analysis. Bioinformatics 31:3691–3693. doi:10.1093/bioinformatics/btv421. 26198102 PMC4817141

