# Supplementary material for: Whole-Genome Sequencing and Comparative Genomics Analysis of a Newly Emerged Multidrug-Resistant Klebsiella pneumoniae Isolate of ST967
Source: Microbiol Spectr. 2023 Apr 6;11(3):e04011-22. doi: 10.1128/spectrum.04011-22 (PMC10269624; doi:10.1128/spectrum.04011-22)
Supplement: Supplemental file 1 — Figures S1-S4. Download spectrum.04011-22-s001.pdf, PDF file, 0.6 MB [file spectrum.04011-22-s001.pdf]

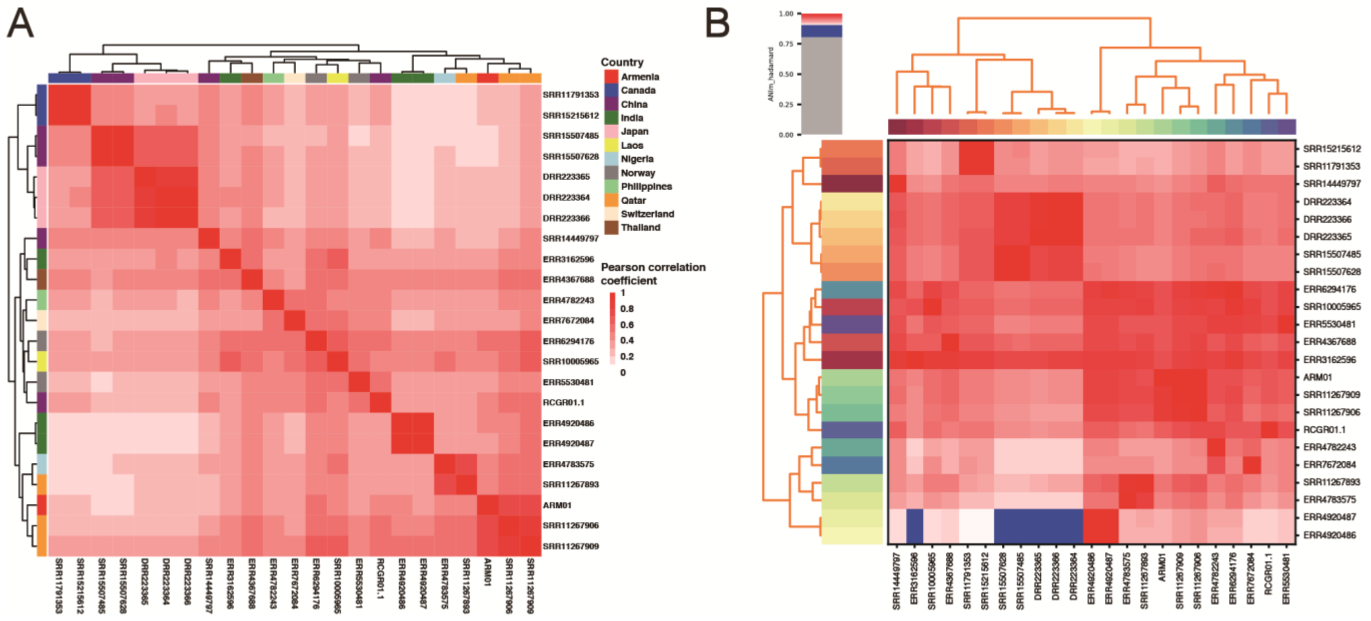

Figure S1. Comparative analysis of the genome composition of 23 *K. pneumoniae* isolates. (A) Comparison on the accessory genes profile based on absence (0) or presence (1) matrix. The heatmap shows the accessory genes correlation for all isolates. The top and side dendrograms indicate the hierarchical clustering by accessory gene content. The colour of the annotation bar represents the country where the isolate was collected. (B) Hierarchical clustering in two dimensions of pairwise ANI comparison of all *K. pneumoniae* isolates. ANI values were generated from Hadamard product matrices of pairwise alignment coverage, total alignment lengths, similarity errors, and percentage identity based on the whole genome sequence.

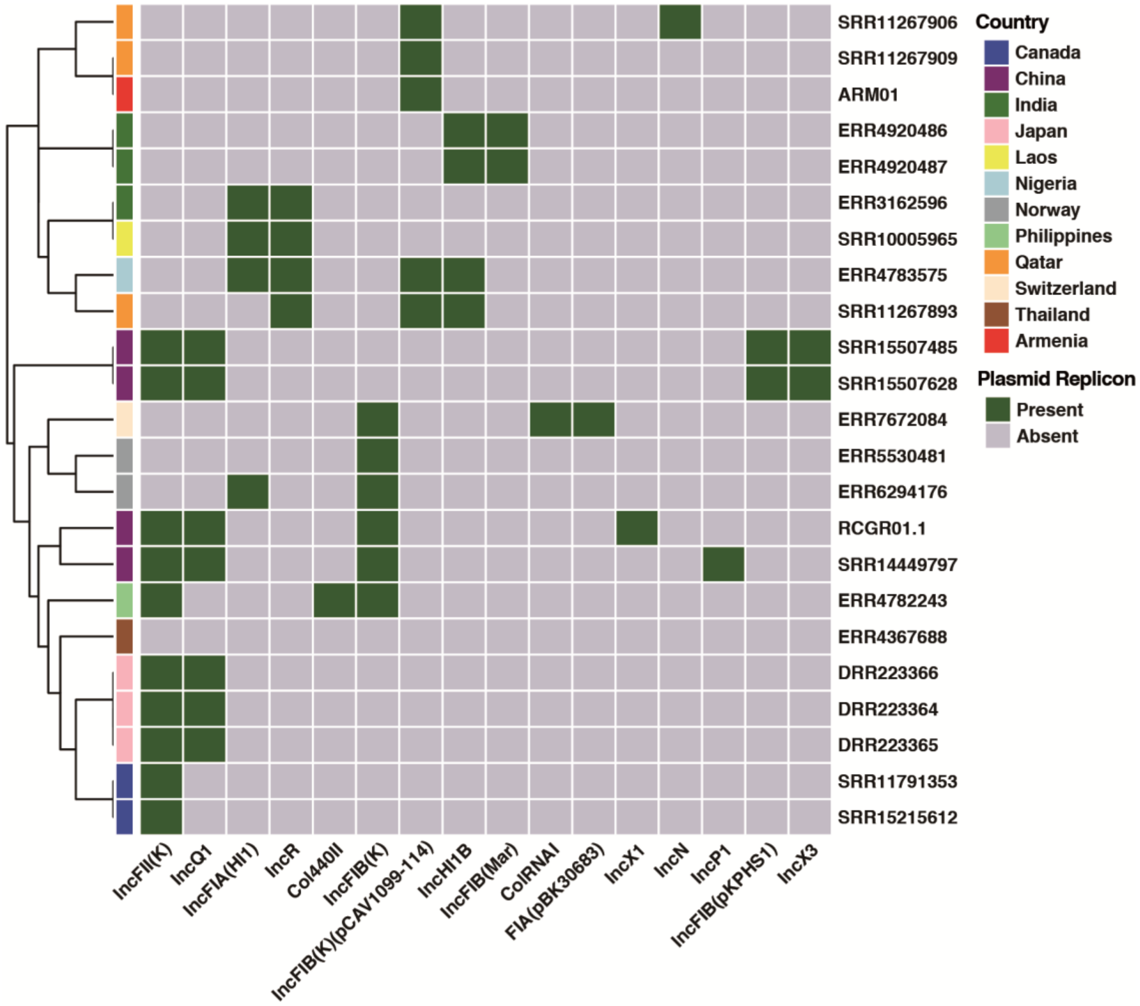

Figure S2. The plasmid replicon profiles of 23 *K. pneumoniae* isolates (dark green: present, grey: absent). The side dendrogram indicates the hierarchical clustering by plasmid replicons content. The colour of the annotation bar represents the country where the isolate was collected.

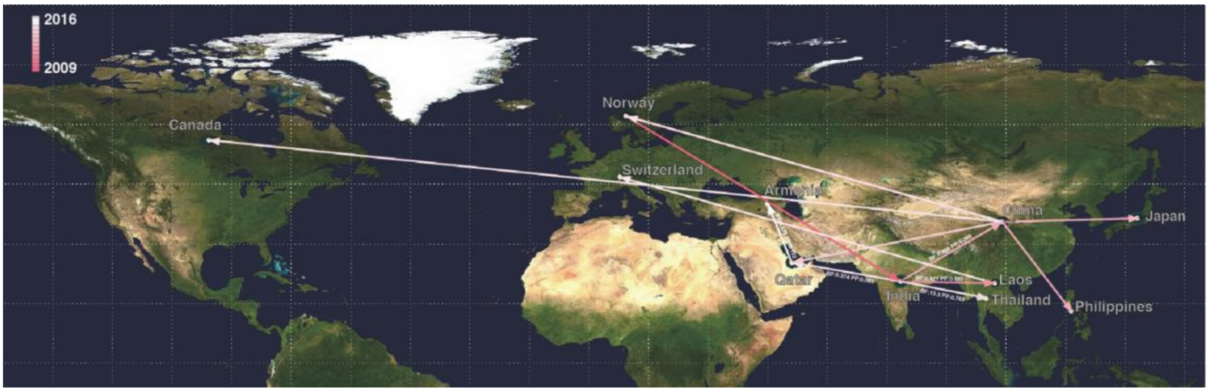

Figure S3. Spatial phylogenetic reconstruction of *K. pneumoniae* ST967 transmission dynamics. The line colour represents transmission date.

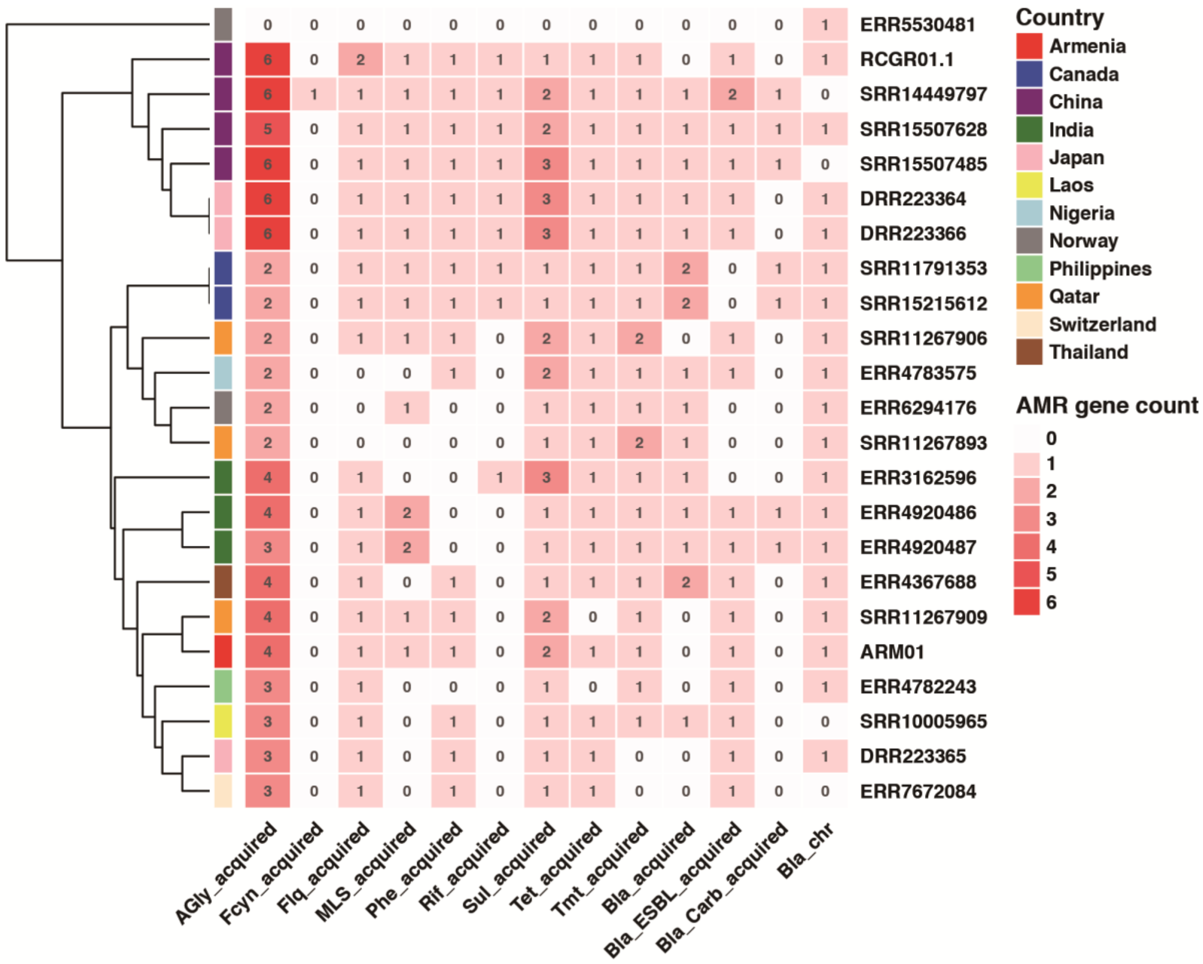

Figure S4. Count of AMR genes associated with each antibiotic. The side dendrogram indicates the hierarchical clustering by AMR gene count. The colour of the annotation bar represents the country where the isolate was collected.
